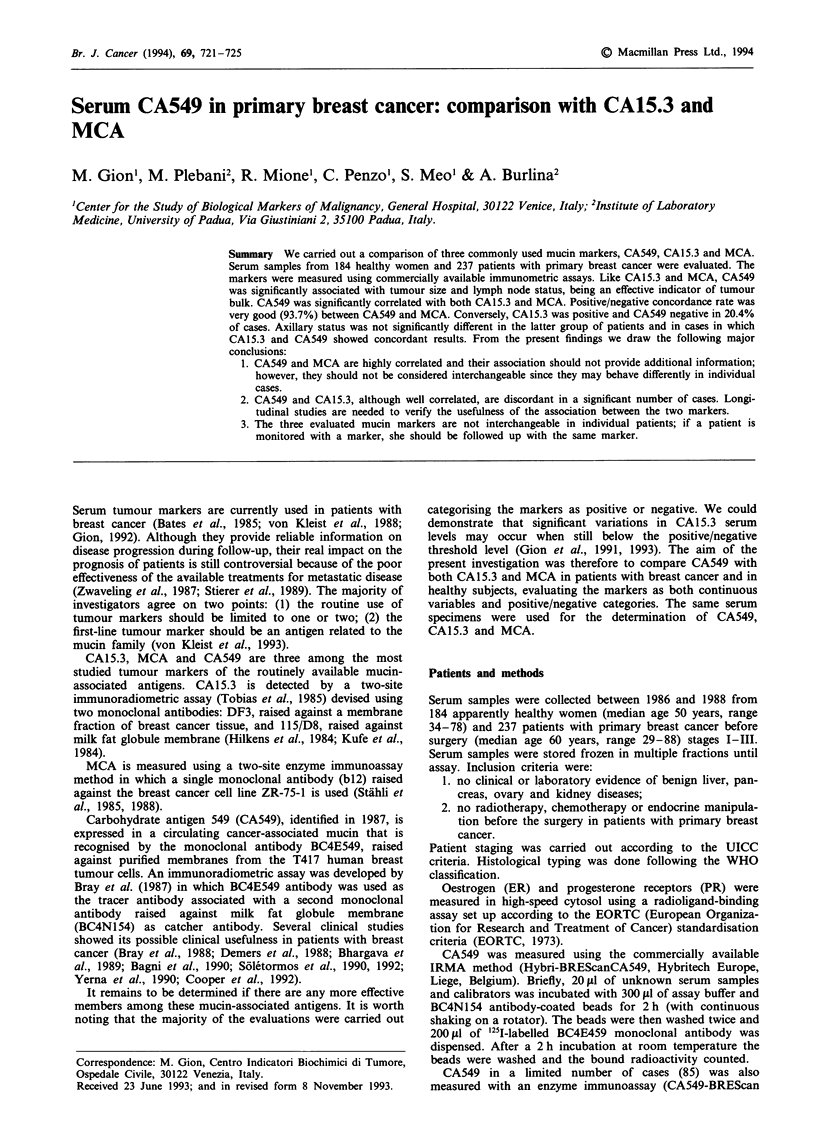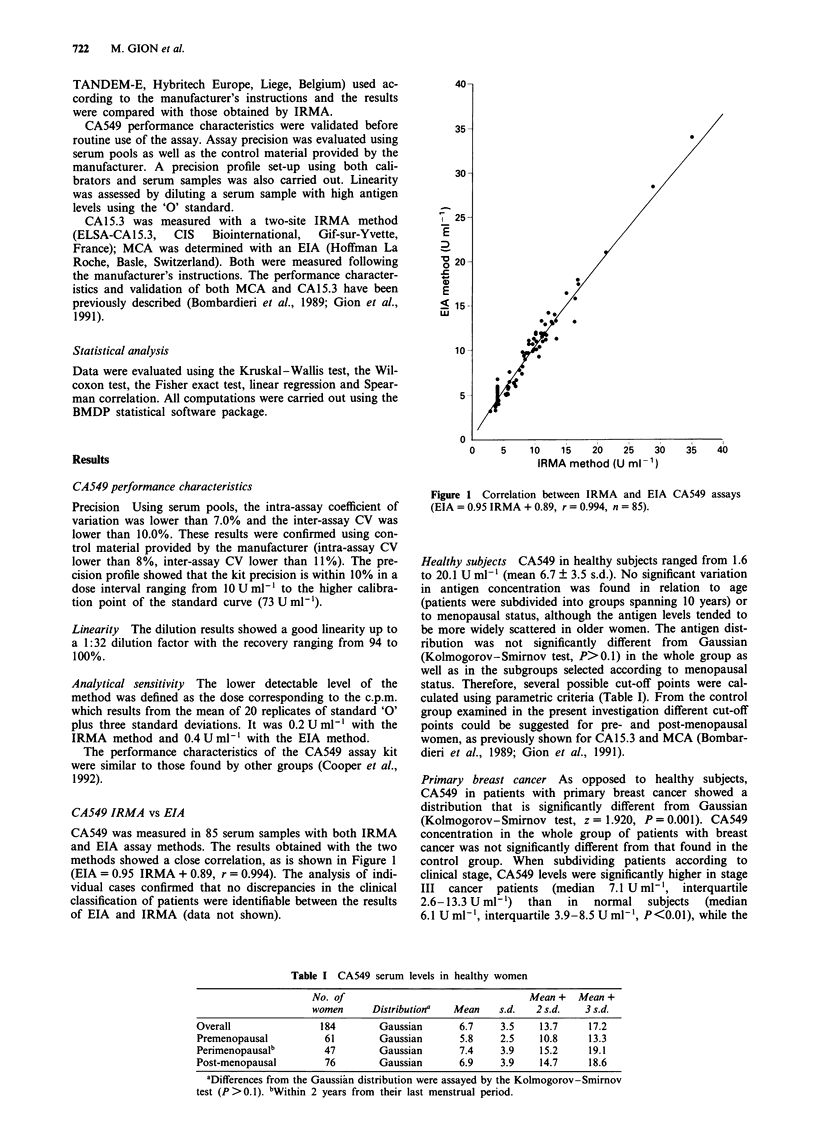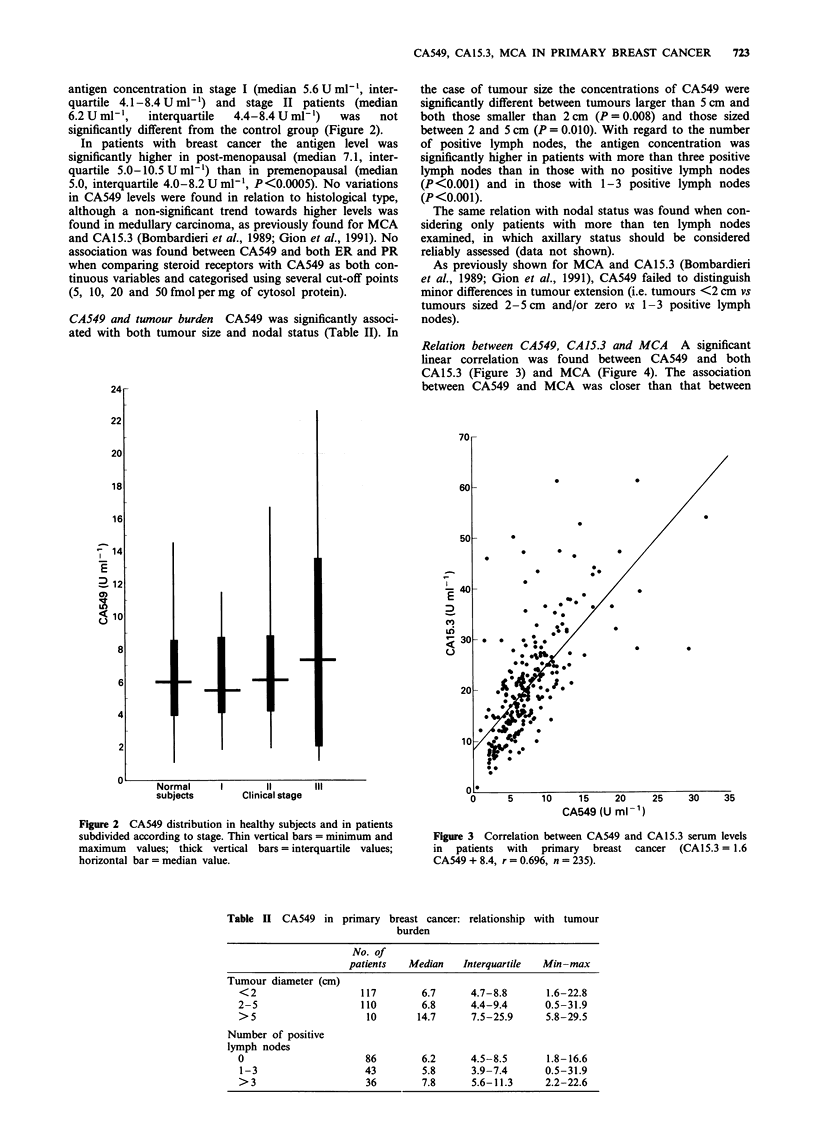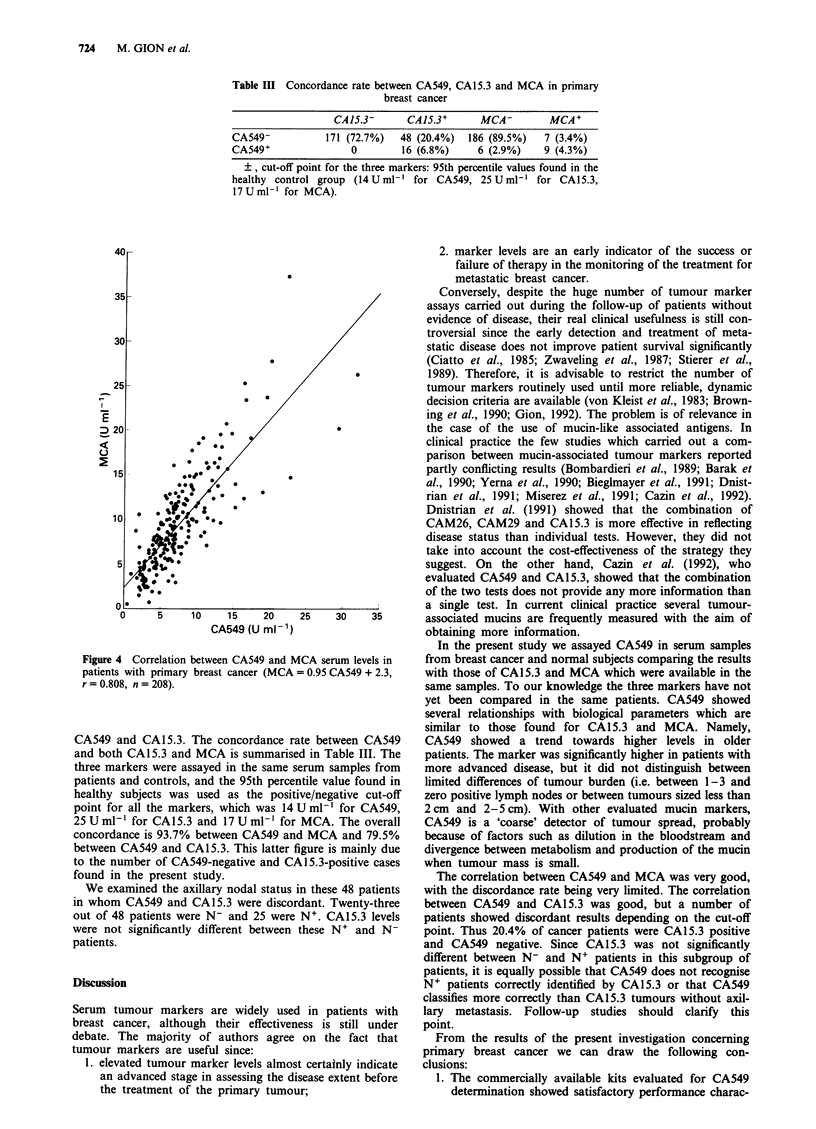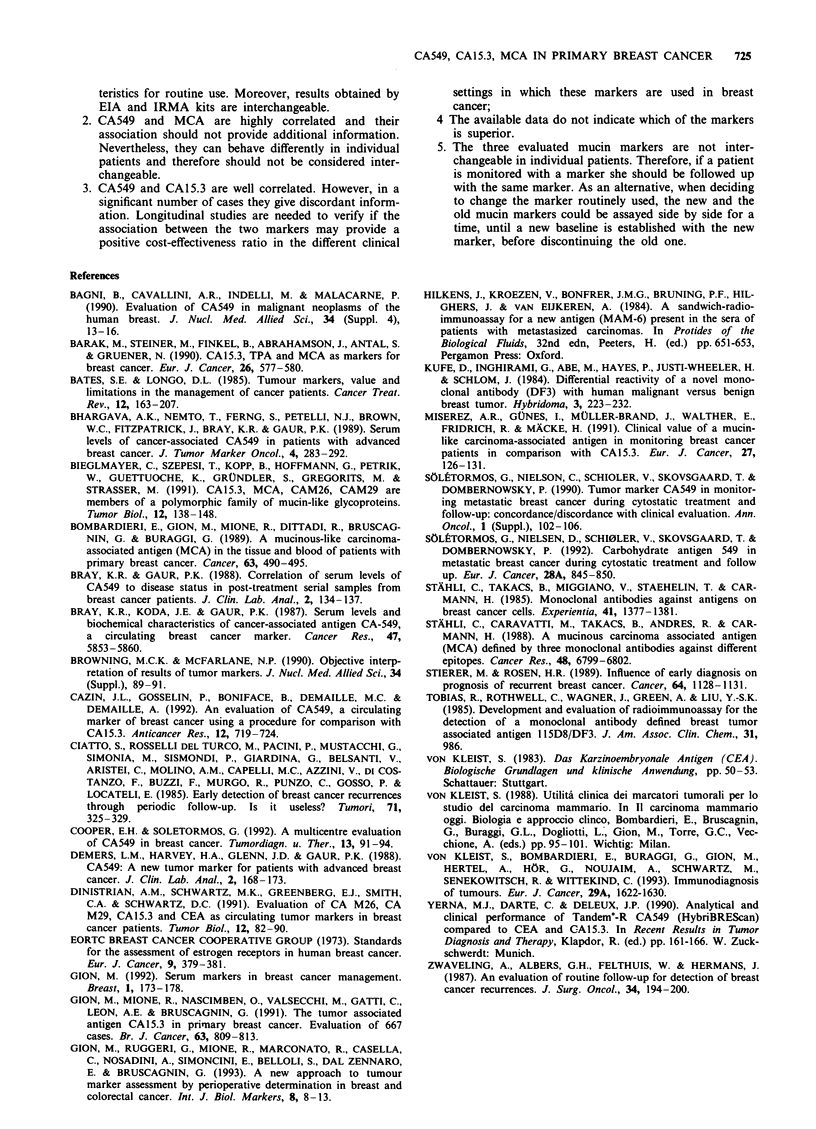# Serum CA549 in primary breast cancer: comparison with CA15.3 and MCA.

**DOI:** 10.1038/bjc.1994.136

**Published:** 1994-04

**Authors:** M. Gion, M. Plebani, R. Mione, C. Penzo, S. Meo, A. Burlina

**Affiliations:** Center for the Study of Biological Markers of Malignancy, General Hospital, Venice, Italy.

## Abstract

We carried out a comparison of three commonly used mucin markers, CA549, CA15.3 and MCA. Serum samples from 184 healthy women and 237 patients with primary breast cancer were evaluated. The markers were measured using commercially available immunometric assays. Like CA15.3 and MCA, CA549 was significantly associated with tumour size and lymph node status, being an effective indicator of tumour bulk. CA549 was significantly correlated with both CA15.3 and MCA. Positive/negative concordance rate was very good (93.7%) between CA549 and MCA. Conversely, CA15.3 was positive and CA549 negative in 20.4% of cases. Axillary status was not significantly different in the latter group of patients and in cases in which CA15.3 and CA549 showed concordant results. From the present findings we draw the following major conclusions: 1. CA549 and MCA are highly correlated and their association should not provide additional information; however, they should not be considered interchangeable since they may behave differently in individual cases. 2. CA549 and CA15.3, although well correlated, are discordant in a significant number of cases. Longitudinal studies are needed to verify the usefulness of the association between the two markers. 3. The three evaluated mucin markers are not interchangeable in individual patients; if a patient is monitored with a marker, she should be followed up with the same marker.


Br. J. Cancer (1994), 69, 721–725 © Macmillan Press Ltd., 1994

### Serum CA549 in primary breast cancer: comparison with CA15.3 and MCA

M. Gion[1], M. Plebani[2], R. Mione[1], C. Penzo[1], S. Meo[1] & A. Burlina[2]

[1]*Center for the Study of Biological Markers of Malignancy, General Hospital, 30122 Venice, Italy;* [2]*Institute of Laboratory Medicine, University of Padua, Via Giustiniani 2, 35100 Padua, Italy.*

**Summary** We carried out a comparison of three commonly used mucin markers, CA549, CA15.3 and MCA. Serum samples from 184 healthy women and 237 patients with primary breast cancer were evaluated. The markers were measured using commercially available immunometric assays. Like CA15.3 and MCA, CA549 was significantly associated with tumour size and lymph node status, being an effective indicator of tumour bulk. CA549 was significantly correlated with both CA15.3 and MCA. Positive/negative concordance rate was very good (93.7%) between CA549 and MCA. Conversely, CA15.3 was positive and CA549 negative in 20.4% of cases. Axillary status was not significantly different in the latter group of patients and in cases in which CA15.3 and CA549 showed concordant results. From the present findings we draw the following major conclusions:

1. CA549 and MCA are highly correlated and their association should not provide additional information; however, they should not be considered interchangeable since they may behave differently in individual cases.
2. CA549 and CA15.3, although well correlated, are discordant in a significant number of cases. Longitudinal studies are needed to verify the usefulness of the association between the two markers.
3. The three evaluated mucin markers are not interchangeable in individual patients; if a patient is monitored with a marker, she should be followed up with the same marker.

Serum tumour markers are currently used in patients with breast cancer (Bates *et al.*, 1985; von Kleist *et al.*, 1988; Gion, 1992). Although they provide reliable information on disease progression during follow-up, their real impact on the prognosis of patients is still controversial because of the poor effectiveness of the available treatments for metastatic disease (Zwaveling *et al.*, 1987; Stierer *et al.*, 1989). The majority of investigators agree on two points: (1) the routine use of tumour markers should be limited to one or two; (2) the first-line tumour marker should be an antigen related to the mucin family (von Kleist *et al.*, 1993).

CA15.3, MCA and CA549 are three among the most studied tumour markers of the routinely available mucin-associated antigens. CA15.3 is detected by a two-site immunoradiometric assay (Tobias *et al.*, 1985) devised using two monoclonal antibodies: DF3, raised against a membrane fraction of breast cancer tissue, and 115/D8, raised against milk fat globule membrane (Hilkens *et al.*, 1984; Kufe *et al.*, 1984).

MCA is measured using a two-site enzyme immunoassay method in which a single monoclonal antibody (b12) raised against the breast cancer cell line ZR-75-1 is used (Stähli *et al.*, 1985, 1988).

Carbohydrate antigen 549 (CA549), identified in 1987, is expressed in a circulating cancer-associated mucin that is recognised by the monoclonal antibody BC4E549, raised against purified membranes from the T417 human breast tumour cells. An immunoradiometric assay was developed by Bray *et al.* (1987) in which BC4E549 antibody was used as the tracer antibody associated with a second monoclonal antibody raised against milk fat globule membrane (BC4N154) as catcher antibody. Several clinical studies showed its possible clinical usefulness in patients with breast cancer (Bray *et al.*, 1988; Demers *et al.*, 1988; Bhargava *et al.*, 1989; Bagni *et al.*, 1990; Sölétormos *et al.*, 1990, 1992; Yerna *et al.*, 1990; Cooper *et al.*, 1992).

It remains to be determined if there are any more effective members among these mucin-associated antigens. It is worth noting that the majority of the evaluations were carried out categorising the markers as positive or negative. We could demonstrate that significant variations in CA15.3 serum levels may occur when still below the positive/negative threshold level (Gion *et al.*, 1991, 1993). The aim of the present investigation was therefore to compare CA549 with both CA15.3 and MCA in patients with breast cancer and in healthy subjects, evaluating the markers as both continuous variables and positive/negative categories. The same serum specimens were used for the determination of CA549, CA15.3 and MCA.

### Patients and methods

Serum samples were collected between 1986 and 1988 from 184 apparently healthy women (median age 50 years, range 34–78) and 237 patients with primary breast cancer before surgery (median age 60 years, range 29–88) stages I–III. Serum samples were stored frozen in multiple fractions until assay. Inclusion criteria were:

1. no clinical or laboratory evidence of benign liver, pancreas, ovary and kidney diseases;
2. no radiotherapy, chemotherapy or endocrine manipulation before the surgery in patients with primary breast cancer.

Patient staging was carried out according to the UICC criteria. Histological typing was done following the WHO classification.

Oestrogen (ER) and progesterone receptors (PR) were measured in high-speed cytosol using a radioligand-binding assay set up according to the EORTC (European Organization for Research and Treatment of Cancer) standardisation criteria (EORTC, 1973).

CA549 was measured using the commercially available IRMA method (Hybri-BREScanCA549, Hybritech Europe, Liege, Belgium). Briefly, 20 µl of unknown serum samples and calibrators was incubated with 300 µl of assay buffer and BC4N154 antibody-coated beads for 2 h (with continuous shaking on a rotator). The beads were then washed twice and 200 µl of $^{125}$I-labelled BC4E459 monoclonal antibody was dispensed. After a 2 h incubation at room temperature the beads were washed and the bound radioactivity counted.

CA549 in a limited number of cases (85) was also measured with an enzyme immunoassay (CA549-BREScan

Correspondence: M. Gion, Centro Indicatori Biochimici di Tumore, Ospedale Civile, 30122 Venezia, Italy.
Received 23 June 1993; and in revised form 8 November 1993.

TANDEM-E, Hybritech Europe, Liege, Belgium) used according to the manufacturer's instructions and the results were compared with those obtained by IRMA.

CA549 performance characteristics were validated before routine use of the assay. Assay precision was evaluated using serum pools as well as the control material provided by the manufacturer. A precision profile set-up using both calibrators and serum samples was also carried out. Linearity was assessed by diluting a serum sample with high antigen levels using the 'O' standard.

CA15.3 was measured with a two-site IRMA method (ELSA-CA15.3, CIS Biointernational, Gif-sur-Yvette, France); MCA was determined with an EIA (Hoffman La Roche, Basle, Switzerland). Both were measured following the manufacturer's instructions. The performance characteristics and validation of both MCA and CA15.3 have been previously described (Bombardieri et al., 1989; Gion et al., 1991).

*Statistical analysis*

Data were evaluated using the Kruskal-Wallis test, the Wilcoxon test, the Fisher exact test, linear regression and Spearman correlation. All computations were carried out using the BMDP statistical software package.

### Results

*CA549 performance characteristics*

*Precision* Using serum pools, the intra-assay coefficient of variation was lower than 7.0% and the inter-assay CV was lower than 10.0%. These results were confirmed using control material provided by the manufacturer (intra-assay CV lower than 8%, inter-assay CV lower than 11%). The precision profile showed that the kit precision is within 10% in a dose interval ranging from 10 U $ml^{-1}$ to the higher calibration point of the standard curve (73 U $ml^{-1}$).

*Linearity* The dilution results showed a good linearity up to a 1:32 dilution factor with the recovery ranging from 94 to 100%.

*Analytical sensitivity* The lower detectable level of the method was defined as the dose corresponding to the c.p.m. which results from the mean of 20 replicates of standard 'O' plus three standard deviations. It was 0.2 U $ml^{-1}$ with the IRMA method and 0.4 U $ml^{-1}$ with the EIA method.

The performance characteristics of the CA549 assay kit were similar to those found by other groups (Cooper et al., 1992).

*CA549 IRMA vs EIA*

CA549 was measured in 85 serum samples with both IRMA and EIA assay methods. The results obtained with the two methods showed a close correlation, as is shown in Figure 1 (EIA = 0.95 IRMA + 0.89, $r = 0.994$). The analysis of individual cases confirmed that no discrepancies in the clinical classification of patients were identifiable between the results of EIA and IRMA (data not shown).

**Figure 1** Correlation between IRMA and EIA CA549 assays (EIA = 0.95 IRMA + 0.89, $r = 0.994$, $n = 85$).

*Healthy subjects* CA549 in healthy subjects ranged from 1.6 to 20.1 U $ml^{-1}$ (mean 6.7 ± 3.5 s.d.). No significant variation in antigen concentration was found in relation to age (patients were subdivided into groups spanning 10 years) or to menopausal status, although the antigen levels tended to be more widely scattered in older women. The antigen distribution was not significantly different from Gaussian (Kolmogorov-Smirnov test, $P > 0.1$) in the whole group as well as in the subgroups selected according to menopausal status. Therefore, several possible cut-off points were calculated using parametric criteria (Table I). From the control group examined in the present investigation different cut-off points could be suggested for pre- and post-menopausal women, as previously shown for CA15.3 and MCA (Bombardieri et al., 1989; Gion et al., 1991).

*Primary breast cancer* As opposed to healthy subjects, CA549 in patients with primary breast cancer showed a distribution that is significantly different from Gaussian (Kolmogorov-Smirnov test, $z = 1.920$, $P = 0.001$). CA549 concentration in the whole group of patients with breast cancer was not significantly different from that found in the control group. When subdividing patients according to clinical stage, CA549 levels were significantly higher in stage III cancer patients (median 7.1 U $ml^{-1}$, interquartile 2.6-13.3 U $ml^{-1}$) than in normal subjects (median 6.1 U $ml^{-1}$, interquartile 3.9-8.5 U $ml^{-1}$, $P < 0.01$), while the

**Table I** CA549 serum levels in healthy women

| | *No. of women* | *Distribution*[a] | *Mean* | *s.d.* | *Mean + 2 s.d.* | *Mean + 3 s.d.* |
|---|---|---|---|---|---|---|
| Overall | 184 | Gaussian | 6.7 | 3.5 | 13.7 | 17.2 |
| Premenopausal | 61 | Gaussian | 5.8 | 2.5 | 10.8 | 13.3 |
| Perimenopausal[b] | 47 | Gaussian | 7.4 | 3.9 | 15.2 | 19.1 |
| Post-menopausal | 76 | Gaussian | 6.9 | 3.9 | 14.7 | 18.6 |

[a]Differences from the Gaussian distribution were assayed by the Kolmogorov-Smirnov test ($P > 0.1$). [b]Within 2 years from their last menstrual period.

antigen concentration in stage I (median 5.6 U $ml^{-1}$, interquartile 4.1–8.4 U $ml^{-1}$) and stage II patients (median 6.2 U $ml^{-1}$, interquartile 4.4–8.4 U $ml^{-1}$) was not significantly different from the control group (Figure 2).

In patients with breast cancer the antigen level was significantly higher in post-menopausal (median 7.1, interquartile 5.0–10.5 U $ml^{-1}$) than in premenopausal (median 5.0, interquartile 4.0–8.2 U $ml^{-1}$, $P<0.0005$). No variations in CA549 levels were found in relation to histological type, although a non-significant trend towards higher levels was found in medullary carcinoma, as previously found for MCA and CA15.3 (Bombardieri *et al.*, 1989; Gion *et al.*, 1991). No association was found between CA549 and both ER and PR when comparing steroid receptors with CA549 as both continuous variables and categorised using several cut-off points (5, 10, 20 and 50 fmol per mg of cytosol protein).

*CA549 and tumour burden* CA549 was significantly associated with both tumour size and nodal status (Table II). In the case of tumour size the concentrations of CA549 were significantly different between tumours larger than 5 cm and both those smaller than 2 cm ($P = 0.008$) and those sized between 2 and 5 cm ($P = 0.010$). With regard to the number of positive lymph nodes, the antigen concentration was significantly higher in patients with more than three positive lymph nodes than in those with no positive lymph nodes ($P<0.001$) and in those with 1–3 positive lymph nodes ($P<0.001$).

The same relation with nodal status was found when considering only patients with more than ten lymph nodes examined, in which axillary status should be considered reliably assessed (data not shown).

As previously shown for MCA and CA15.3 (Bombardieri *et al.*, 1989; Gion *et al.*, 1991), CA549 failed to distinguish minor differences in tumour extension (i.e. tumours <2 cm *vs* tumours sized 2–5 cm and/or zero *vs* 1–3 positive lymph nodes).

*Relation between CA549, CA15.3 and MCA* A significant linear correlation was found between CA549 and both CA15.3 (Figure 3) and MCA (Figure 4). The association between CA549 and MCA was closer than that between

**Figure 2** CA549 distribution in healthy subjects and in patients subdivided according to stage. Thin vertical bars = minimum and maximum values; thick vertical bars = interquartile values; horizontal bar = median value.

**Figure 3** Correlation between CA549 and CA15.3 serum levels in patients with primary breast cancer (CA15.3 = 1.6 CA549 + 8.4, $r = 0.696$, $n = 235$).

**Table II** CA549 in primary breast cancer: relationship with tumour burden

| | *No. of patients* | *Median* | *Interquartile* | *Min–max* |
|---|---|---|---|---|
| Tumour diameter (cm) | | | | |
| <2 | 117 | 6.7 | 4.7–8.8 | 1.6–22.8 |
| 2–5 | 110 | 6.8 | 4.4–9.4 | 0.5–31.9 |
| >5 | 10 | 14.7 | 7.5–25.9 | 5.8–29.5 |
| Number of positive lymph nodes | | | | |
| 0 | 86 | 6.2 | 4.5–8.5 | 1.8–16.6 |
| 1–3 | 43 | 5.8 | 3.9–7.4 | 0.5–31.9 |
| >3 | 36 | 7.8 | 5.6–11.3 | 2.2–22.6 |

Table III Concordance rate between CA549, CA15.3 and MCA in primary breast cancer

| | *CA15.3⁻* | *CA15.3⁺* | *MCA⁻* | *MCA⁺* |
|---|---|---|---|---|
| $CA549^-$ | 171 (72.7%) | 48 (20.4%) | 186 (89.5%) | 7 (3.4%) |
| $CA549^+$ | 0 | 16 (6.8%) | 6 (2.9%) | 9 (4.3%) |

±, cut-off point for the three markers: 95th percentile values found in the healthy control group ($14\,U\,ml^{-1}$ for CA549, $25\,U\,ml^{-1}$ for CA15.3, $17\,U\,ml^{-1}$ for MCA).

**Figure 4** Correlation between CA549 and MCA serum levels in patients with primary breast cancer (MCA = 0.95 CA549 + 2.3, $r = 0.808$, $n = 208$).

CA549 and CA15.3. The concordance rate between CA549 and both CA15.3 and MCA is summarised in Table III. The three markers were assayed in the same serum samples from patients and controls, and the 95th percentile value found in healthy subjects was used as the positive/negative cut-off point for all the markers, which was $14\,U\,ml^{-1}$ for CA549, $25\,U\,ml^{-1}$ for CA15.3 and $17\,U\,ml^{-1}$ for MCA. The overall concordance is 93.7% between CA549 and MCA and 79.5% between CA549 and CA15.3. This latter figure is mainly due to the number of CA549-negative and CA15.3-positive cases found in the present study.

We examined the axillary nodal status in these 48 patients in whom CA549 and CA15.3 were discordant. Twenty-three out of 48 patients were $N^-$ and 25 were $N^+$. CA15.3 levels were not significantly different between these $N^+$ and $N^-$ patients.

### Discussion

Serum tumour markers are widely used in patients with breast cancer, although their effectiveness is still under debate. The majority of authors agree on the fact that tumour markers are useful since:

1. elevated tumour marker levels almost certainly indicate an advanced stage in assessing the disease extent before the treatment of the primary tumour;
2. marker levels are an early indicator of the success or failure of therapy in the monitoring of the treatment for metastatic breast cancer.

Conversely, despite the huge number of tumour marker assays carried out during the follow-up of patients without evidence of disease, their real clinical usefulness is still controversial since the early detection and treatment of metastatic disease does not improve patient survival significantly (Ciatto *et al.*, 1985; Zwaveling *et al.*, 1987; Stierer *et al.*, 1989). Therefore, it is advisable to restrict the number of tumour markers routinely used until more reliable, dynamic decision criteria are available (von Kleist *et al.*, 1983; Browning *et al.*, 1990; Gion, 1992). The problem is of relevance in the case of the use of mucin-like associated antigens. In clinical practice the few studies which carried out a comparison between mucin-associated tumour markers reported partly conflicting results (Bombardieri *et al.*, 1989; Barak *et al.*, 1990; Yerna *et al.*, 1990; Bieglmayer *et al.*, 1991; Dnistrian *et al.*, 1991; Miserez *et al.*, 1991; Cazin *et al.*, 1992). Dnistrian *et al.* (1991) showed that the combination of CAM26, CAM29 and CA15.3 is more effective in reflecting disease status than individual tests. However, they did not take into account the cost-effectiveness of the strategy they suggest. On the other hand, Cazin *et al.* (1992), who evaluated CA549 and CA15.3, showed that the combination of the two tests does not provide any more information than a single test. In current clinical practice several tumour-associated mucins are frequently measured with the aim of obtaining more information.

In the present study we assayed CA549 in serum samples from breast cancer and normal subjects comparing the results with those of CA15.3 and MCA which were available in the same samples. To our knowledge the three markers have not yet been compared in the same patients. CA549 showed several relationships with biological parameters which are similar to those found for CA15.3 and MCA. Namely, CA549 showed a trend towards higher levels in older patients. The marker was significantly higher in patients with more advanced disease, but it did not distinguish between limited differences of tumour burden (i.e. between 1–3 and zero positive lymph nodes or between tumours sized less than 2 cm and 2–5 cm). With other evaluated mucin markers, CA549 is a 'coarse' detector of tumour spread, probably because of factors such as dilution in the bloodstream and divergence between metabolism and production of the mucin when tumour mass is small.

The correlation between CA549 and MCA was very good, with the discordance rate being very limited. The correlation between CA549 and CA15.3 was good, but a number of patients showed discordant results depending on the cut-off point. Thus 20.4% of cancer patients were CA15.3 positive and CA549 negative. Since CA15.3 was not significantly different between $N^-$ and $N^+$ patients in this subgroup of patients, it is equally possible that CA549 does not recognise $N^+$ patients correctly identified by CA15.3 or that CA549 classifies more correctly than CA15.3 tumours without axillary metastasis. Follow-up studies should clarify this point.

From the results of the present investigation concerning primary breast cancer we can draw the following conclusions:

1. The commercially available kits evaluated for CA549 determination showed satisfactory performance characteristics for routine use. Moreover, results obtained by EIA and IRMA kits are interchangeable.

2. CA549 and MCA are highly correlated and their association should not provide additional information. Nevertheless, they can behave differently in individual patients and therefore should not be considered interchangeable.
3. CA549 and CA15.3 are well correlated. However, in a significant number of cases they give discordant information. Longitudinal studies are needed to verify if the association between the two markers may provide a positive cost-effectiveness ratio in the different clinical settings in which these markers are used in breast cancer;
4 The available data do not indicate which of the markers is superior.
5. The three evaluated mucin markers are not interchangeable in individual patients. Therefore, if a patient is monitored with a marker she should be followed up with the same marker. As an alternative, when deciding to change the marker routinely used, the new and the old mucin markers could be assayed side by side for a time, until a new baseline is established with the new marker, before discontinuing the old one.

### References

BAGNI, B., CAVALLINI, A.R., INDELLI, M. & MALACARNE, P. (1990). Evaluation of CA549 in malignant neoplasms of the human breast. *J. Nucl. Med. Allied Sci.*, **34** (Suppl. 4), 13–16.

BARAK, M., STEINER, M., FINKEL, B., ABRAHAMSON, J., ANTAL, S. & GRUENER, N. (1990). CA15.3, TPA and MCA as markers for breast cancer. *Eur. J. Cancer*, **26**, 577–580.

BATES, S.E. & LONGO, D.L. (1985). Tumour markers, value and limitations in the management of cancer patients. *Cancer Treat. Rev.*, **12**, 163–207.

BHARGAVA, A.K., NEMTO, T., FERNG, S., PETELLI, N.J., BROWN, W.C., FITZPATRICK, J., BRAY, K.R. & GAUR, P.K. (1989). Serum levels of cancer-associated CA549 in patients with advanced breast cancer. *J. Tumor Marker Oncol.*, **4**, 283–292.

BIEGLMAYER, C., SZEPESI, T., KOPP, B., HOFFMANN, G., PETRIK, W., GUETTUOCHE, K., GRÜNDLER, S., GREGORITS, M. & STRASSER, M. (1991). CA15.3, MCA, CAM26, CAM29 are members of a polymorphic family of mucin-like glycoproteins. *Tumor Biol.*, **12**, 138–148.

BOMBARDIERI, E., GION, M., MIONE, R., DITTADI, R., BRUSCAGNIN, G. & BURAGGI, G. (1989). A mucinous-like carcinoma-associated antigen (MCA) in the tissue and blood of patients with primary breast cancer. *Cancer*, **63**, 490–495.

BRAY, K.R. & GAUR, P.K. (1988). Correlation of serum levels of CA549 to disease status in post-treatment serial samples from breast cancer patients. *J. Clin. Lab. Anal.*, **2**, 134–137.

BRAY, K.R., KODA, J.E. & GAUR, P.K. (1987). Serum levels and biochemical characteristics of cancer-associated antigen CA-549, a circulating breast cancer marker. *Cancer Res.*, **47**, 5853–5860.

BROWNING, M.C.K. & MCFARLANE, N.P. (1990). Objective interpretation of results of tumor markers. *J. Nucl. Med. Allied Sci.*, **34** (Suppl.), 89–91.

CAZIN, J.L., GOSSELIN, P., BONIFACE, B., DEMAILLE, M.C. & DEMAILLE, A. (1992). An evaluation of CA549, a circulating marker of breast cancer using a procedure for comparison with CA15.3. *Anticancer Res.*, **12**, 719–724.

CIATTO, S., ROSSELLI DEL TURCO, M., PACINI, P., MUSTACCHI, G., SIMONIA, M., SISMONDI, P., GIARDINA, G., BELSANTI, V., ARISTEI, C., MOLINO, A.M., CAPELLI, M.C., AZZINI, V., DI COSTANZO, F., BUZZI, F., MURGO, R., PUNZO, C., GOSSO, P. & LOCATELI, E. (1985). Early detection of breast cancer recurrences through periodic follow-up. Is it useless? *Tumori*, **71**, 325–329.

COOPER, E.H. & SOLETORMOS, G. (1992). A multicentre evaluation of CA549 in breast cancer. *Tumordiagn. u. Ther.*, **13**, 91–94.

DEMERS, L.M., HARVEY, H.A., GLENN, J.D. & GAUR, P.K. (1988). CA549: A new tumor marker for patients with advanced breast cancer. *J. Clin. Lab. Anal.*, **2**, 168–173.

DINISTRIAN, A.M., SCHWARTZ, M.K., GREENBERG, E.J., SMITH, C.A. & SCHWARTZ, D.C. (1991). Evaluation of CA M26, CA M29, CA15.3 and CEA as circulating tumor markers in breast cancer patients. *Tumor Biol.*, **12**, 82–90.

EORTC BREAST CANCER COOPERATIVE GROUP (1973). Standards for the assessment of estrogen receptors in human breast cancer. *Eur. J. Cancer*, **9**, 379–381.

GION, M. (1992). Serum markers in breast cancer management. *Breast*, **1**, 173–178.

GION, M., MIONE, R., NASCIMBEN, O., VALSECCHI, M., GATTI, C., LEON, A.E. & BRUSCAGNIN, G. (1991). The tumor associated antigen CA15.3 in primary breast cancer. Evaluation of 667 cases. *Br. J. Cancer*, **63**, 809–813.

GION, M., RUGGERI, G., MIONE, R., MARCONATO, R., CASELLA, C., NOSADINI, A., SIMONCINI, E., BELLOLI, S., DAL ZENNARO, E. & BRUSCAGNIN, G. (1993). A new approach to tumour marker assessment by perioperative determination in breast and colorectal cancer. *Int. J. Biol. Markers*, **8**, 8–13.

HILKENS, J., KROEZEN, V., BONFRER, J.M.G., BRUNING, P.F., HILGHERS, J. & VAN EIJKEREN, A. (1984). A sandwich-radioimmunoassay for a new antigen (MAM-6) present in the sera of patients with metastasized carcinomas. In *Protides of the Biological Fluids*, 32nd edn, Peeters, H. (ed.) pp. 651-653, Pergamon Press: Oxford.

KUFE, D., INGHIRAMI, G., ABE, M., HAYES, P., JUSTI-WHEELER, H. & SCHLOM, J. (1984). Differential reactivity of a novel monoclonal antibody (DF3) with human malignant versus benign breast tumor. *Hybridoma*, **3**, 223–232.

MISEREZ, A.R., GÜNES, I., MÜLLER-BRAND, J., WALTHER, E., FRIDRICH, R. & MÄCKE, H. (1991). Clinical value of a mucin-like carcinoma-associated antigen in monitoring breast cancer patients in comparison with CA15.3. *Eur. J. Cancer*, **27**, 126–131.

SÖLÉTORMOS, G., NIELSON, C., SCHIOLER, V., SKOVSGAARD, T. & DOMBERNOWSKY, P. (1990). Tumor marker CA549 in monitoring metastatic breast cancer during cytostatic treatment and follow-up: concordance/discordance with clinical evaluation. *Ann. Oncol.*, **1** (Suppl.), 102–106.

SÖLÉTORMOS, G., NIELSEN, D., SCHIØLER, V., SKOVSGAARD, T. & DOMBERNOWSKY, P. (1992). Carbohydrate antigen 549 in metastatic breast cancer during cytostatic treatment and follow up. *Eur. J. Cancer*, **28A**, 845–850.

STÄHLI, C., TAKACS, B., MIGGIANO, V., STAEHELIN, T. & CARMANN, H. (1985). Monoclonal antibodies against antigens on breast cancer cells. *Experientia*, **41**, 1377–1381.

STÄHLI, C., CARAVATTI, M., TAKACS, B., ANDRES, R. & CARMANN, H. (1988). A mucinous carcinoma associated antigen (MCA) defined by three monoclonal antibodies against different epitopes. *Cancer Res.*, **48**, 6799–6802.

STIERER, M. & ROSEN, H.R. (1989). Influence of early diagnosis on prognosis of recurrent breast cancer. *Cancer*, **64**, 1128–1131.

TOBIAS, R., ROTHWELL, C., WAGNER, J., GREEN, A. & LIU, Y.-S.K. (1985). Development and evaluation of radioimmunoassay for the detection of a monoclonal antibody defined breast tumor associated antigen 115D8/DF3. *J. Am. Assoc. Clin. Chem.*, **31**, 986.

VON KLEIST, S. (1983). *Das Karzinoembryonale Antigen (CEA). Biologische Grundlagen und klinische Anwendung*, pp. 50–53. Schattauer: Stuttgart.

VON KLEIST, S. (1988). Utilitá clinica dei marcatori tumorali per lo studio del carcinoma mammario. In Il carcinoma mammario oggi. Biologia e approccio clinco, Bombardieri, E., Bruscagnin, G., Buraggi, G.L., Dogliotti, L., Gion, M., Torre, G.C., Vecchione, A. (eds.) pp. 95–101. Wichtig: Milan.

VON KLEIST, S., BOMBARDIERI, E., BURAGGI, G., GION, M., HERTEL, A., HÖR, G., NOUJAIM, A., SCHWARTZ, M., SENEKOWITSCH, R. & WITTEKIND, C. (1993). Immunodiagnosis of tumours. *Eur. J. Cancer*, **29A**, 1622-1630.

YERNA, M.J., DARTE, C. & DELEUX, J.P. (1990). Analytical and clinical performance of Tandem®-R CA549 (HybriBREScan) compared to CEA and CA15.3. In *Recent Results in Tumor Diagnosis and Therapy*, Klapdor, R. (ed.) pp. 161-166. W. Zuckschwerdt: Munich.

ZWAVELING, A., ALBERS, G.H., FELTHUIS, W. & HERMANS, J. (1987). An evaluation of routine follow-up for detection of breast cancer recurrences. *J. Surg. Oncol.*, **34**, 194–200.